# Primary Care Providers Knowledge, Attitude and Practices Related to Hepatitis C Screening and Treatment in the Oral Direct Acting Antiviral Agents Era

**DOI:** 10.4172/2161-0711.1000481

**Published:** 2016-10-28

**Authors:** O Falade-Nwulia, A McAdams-Mahmoud, R Irvin, A Niculescu, KR Page, M Mix, DL Thomas, MS Sulkowski, SH Mehta

**Affiliations:** 1 Johns Hopkins University School of Medicine, Baltimore, USA; 2 Johns Hopkins Bloomberg School of Public Health, Baltimore, USA

**Keywords:** Primary care, Hepatitis C, Treatment

## Abstract

**Background:**

There are over 3 million Americans infected with hepatitis C virus (HCV). Despite recent advances in HCV treatment, a major barrier to care remains a limited number of treaters. HCV therapy provision by primary care providers (PCPs) could expand access by increasing the pool of HCV treating clinicians.

**Objective:**

To characterize current HCV care practices, willingness and self-efficacy of PCPs to become HCV treaters.

**Design, participants and main measures:**

Two hundred and seventy one PCPs were identified from community clinics affiliated with a large academic center and 4 large federally qualified health centers in Baltimore, MD. An internet-based survey was administered to assess provider demographics, clinical practice site and willingness to provide HCV care. Factors associated with willingness to provide HCV care were examined using odds ratios (OR).

**Key results:**

Among 129 (48%) PCPs who responded, the majority (70%) had an MD/DO degree and were white (60%). Only a few PCPs, 12 (10%), had treated at least 1 patient for HCV in the prior year. Although only 22% agreed that HCV treatment should be provided by PCPs, 84% were interested in more HCV training. Willingness to provide treatment was strongly linked to having a high proportion of HCV-infected patients (>20% versus <20%; OR 3.9; 95% confidence interval [CI] 1.5–10) and availability of other services at the primary care site including HIV treatment (OR 6.5; 95% CI 2.5–16.5), substance abuse treatment (OR 3.3; 95% CI 1.3–8.4) and mental health services (OR 4.9; 95% CI 2.0–12.1).

**Conclusion:**

These data suggest that efforts to expand HCV medical provider capacity will be most impactful if they initially focus HCV training on PCPs with a high prevalence of HCV among their patients and existing systems to support HCV care.

## Introduction

There are over 3.2 million Americans infected with the hepatitis C virus (HCV) [1]. HCV infection is associated with liver cirrhosis, liver cancer and death and since 2007 has accounted for more deaths in the US than HIV [2]. Despite the availability of simple treatments with minimal side effects which can cure HCV infection in most individuals in 12 weeks or less, the majority of HCV infected individuals remain undiagnosed and untreated [3].

Primary care clinics have traditionally been considered the principal hepatitis C screening venues in the United States. Rates of HCV screening have, however, historically been low. Data from the National Health and Nutrition Examination Survey 2001–2008 (NHANES) reports that only 3.7% of respondents were tested because they or their doctors thought they were at risk for HCV [3]. Because over 75% of HCV infections are in individuals born between 1945 and 1965, the Centers for Disease Control and Prevention (CDC) and the United States Preventative Services Task Force (USPSTF) have recommended that individuals born in these years (“the birth cohort”) be tested at least once for HCV, independent of any identified risk factors [4,5]. These recommendations alleviate the discomfort reported by clinicians in eliciting HCV risk factors and may increase HCV testing and case detection rates [6]. When fully integrated into primary care, routine “birth cohort” based HCV testing is expected to lead to identification of previously undiagnosed persons with HCV infection [7,8].

Historically, HCV treatment has been provided primarily by hepatology, gastroenterology and infectious disease specialists and not by primary care physicians (PCPs). This has led to significant delays in or failure of HCV care linkage due to a combination of patient and provider level factors including long specialist wait times or lack of willingness by patients to attend HCV care appointments at alternate venues [6,9]. Newly approved and highly effective direct acting antivirals (DAA) make it possible for HCV to be cured with low patient and provider burden and has raised the possibility of HCV elimination in the US [10]. One key challenge to achieving elimination is an insufficient number of providers who can treat HCV. PCPs may hold the key to expanding the pool of HCV treating providers and thus positively impacting the trajectory of the HCV epidemic [10]. In order to support these PCPs, care managers, who are trained liaisons between PCPs and specialists, may also be able to provide support in eliminating the gap between screening and treatment for HCV-positive patients and by delivering care coordination throughout HCV treatment and follow-up [11].

Although several studies have suggested a need for increased HCV-specific education among PCPs [6], there are limited data on PCP knowledge and awareness of HCV infection, testing and treatment. One analysis of public inquiries regarding HCV suggested that the most common queries from health professionals were about serology and transmission [12]. Another survey of PCPs indicated that almost half of all respondents had no experience with HCV infection at all, and more than a quarter did not know how to proceed with managing a hypothetical patient with chronic HCV infection [13]. Additional data indicate a lack of understanding around the CDC recommendations for HCV risk reduction, with a significant percentage of providers counseling patients incorrectly by suggesting condoms and avoiding breastfeeding, while not mentioning reduction of alcohol consumption [14]. Despite these knowledge gaps, even in the prior era of complex interferon-based therapies, it has been demonstrated that community-based PCPs with appropriate training and support can provide HCV treatment and achieve HCV cure rates comparable to those of specialists in academic centers [15,16]. Data from the oral DAA era on PCP knowledge of, self-efficacy and interest in providing HCV treatment are even more limited.

To update current knowledge and help guide development of interventions to increase access to hepatitis C testing and care services, this study aimed to assess current practices, perceived self-efficacy and willingness to provide HCV care among primary care providers in Maryland.

## Materials and Methods

We conducted a cross-sectional survey of primary care providers in care sites within Maryland, USA. The survey was administered via the internet between September 2014 and March 2015.

### Participants

Participants included physicians, nurse practitioners and physician assistant primary care providers (PCPs). PCPs were identified through a listing of providers affiliated with a large academic center (Johns Hopkins Medical Institutions) and four large federally qualified health centers (Chase Braxton Health Systems, Health Care for the Homeless, JAI medical systems and Total Health Care) with practice sites throughout Maryland. Each site sent the survey to their internal provider email contact list.

### Survey

A 42 item quantitative online survey hosted by commercial survey software tool, Survey Monkey, was sent by email to participating providers. The survey comprised of multiple choice answer options, with some write-in response options. The survey collected data on provider demographic and clinical site characteristic, knowledge, attitudes and perceptions of substance abuse and HCV care including self-assessments of substance abuse care proficiency, HCV care proficiency and interest in providing HCV treatment. To ensure confidentiality, participants were consented online and assigned unique ID numbers. The survey was intended to be 15 minutes in length and providers could elect to receive a $10 gift card for their participation. The study was approved by the Johns Hopkins University School of Medicine Institutional Review Board.

### Analysis

Descriptive analyses were used to describe baseline provider demographic and clinical site characteristics, HCV screening and treatment practices, HCV care proficiency and interest in HCV treatment. A composite score was created for PCP perceived self-efficacy based on five questions on aspects of HCV care for a maximum score of 20. PCPs with a score of 15 or greater were classified as having a self-rated proficiency of being skilled in HCV care and those with a score of 10–15 were classified as having a skill level of average among their peers. Assessment of interest in providing HCV care was based on response to the statement “HCV treatment should be provided by PCPs in the all oral HCV DAA era” based on a 5-point Likert scale ranging from strongly disagree to strongly agree. PCPs were considered to be willing to provide HCV treatment if they responded “agree” or “strongly agree” to this statement. We conducted logistic regression analysis to assess key provider and site characteristics associated with HCV treatment. A p-value<0.05 was considered statistically significant.

Of 271 PCPs who received an email invitation, 129 (48%) responded. Nine PCPs did not respond to the statement “HCV treatment should be provided by PCPs in the all oral HCV DAA era” and were excluded from subsequent analysis leaving a total of 120 PCPs for analysis. Six PCPs did not respond to questions used to assess HCV care proficiency but were included for other analysis.

## Results

### Provider and clinical site characteristics

As summarized in Table 1, providers were predominantly female (68%), white (60%) had an MD/DO degree (70%) and had provided primary care to over 500 patients in the preceding year (74%). The primary care sites of these PCPs were largely non-academic community based primary care sites (84%) with an equal proportion located in urban (48%) and non-urban (51%) locations; 21% of PCPs worked at sites where greater than 20% of their patients were HCV infected. A majority (64%) of PCPs agreed that HCV treatment was important in the communities they served while 48% believed that developing capacity to treat hepatitis C would benefit their clinic.

### Current practice behaviors

PCPs reported varying HCV screening practices, with 13% of PCPs reporting rarely screening for HCV, 27% screening in response to clinical factors such as elevated transaminases, 35% screening patients who have HCV risk factors and 25% screening everyone. A majority (78%) of PCPs were aware of the CDC defined “birth cohort” based HCV screening recommendations. These recommendations had been implemented in 60% of these PCPs practice settings with use of an EMR based reminder for HCV screening in 57% of practice settings. A majority of PCPs (89%) reported referring HCV infected patients in their practice for HCV treatment elsewhere with only 56% of PCPs consistently referring (>75%) of their HCV infected patients for treatment. Only 12 PCPs (10%) had treated at least 1 patient for HCV in the preceding year.

### Self-rated HCV care proficiency

Based on responses to questions on self-rated proficiency in HCV care (Table 2), the majority of PCPs reported limited or no knowledge of HCV care. Only 5 (4%) had a score of >15 consistent with perceived self-efficacy of being skilled in HCV care and 36 (26%) a score of >10 consistent with a perceived self-efficacy of average among peers. HCV care proficiency was not associated with provider type (MD/DO *vs.* NP/PA), age, number of years in practice, proportion of patient who were PWID, patient volume or practice site (urban *vs.* non-urban). In contrast, PCPs reported substantially higher alcohol use assessment and care proficiency with 47% of PCPs considering themselves very knowledgeable or expert.

### HCV knowledge

When presented with a clinical case of a patient presenting with a new positive hepatitis C antibody result, 102/120 (85%) appropriately selected HCV RNA testing as the next step in management, however, only 58/120 (44%) correctly identified a need for alcohol use counseling and referral for alcohol use treatment if indicated. The majority 92/120 (77%) of PCPs were aware of the availability of new oral direct acting agents which can cure hepatitis C in 12 weeks or less and most 80/120 (67%) correctly identified HCV genotype as an important part of the HCV treatment decision.

### Hepatitis C care training

One-third of PCPs reported having no prior hepatitis C care training or experience. Among the 71 that reported prior hepatitis C training, this training was received through on-line modules or tutorials (49%), sessions at conference meetings (46%), local CME meeting (37%) and preceptor-ships from another provider (21%). When provided a menu of options through which HCV training could be provided, most (84%) of PCPs indicated one of more avenues through which they would like to receive additional HCV training. Among the 109 PCPs indicating a preference for additional HCV training, 74% noted a preference for online modules, 57%, sessions at conferences, 48% local CME workshops, 26% preceptor-ships with an experienced hepatitis C provider in clinic, and 30% continued discussion with providers treating hepatitis C in their communities.

### Interest in HCV care provision by PCP

Only 22% of PCPs reported agreement with the statement that PCPs should provide HCV treatment in the all oral DAA era. PCPs who had provided care to a higher number (>100 compared to <100 or >1000 compared to <100) patients in the preceding year were significantly less likely to agree with HCV care provision by PCP. Agreement with PCP provision of HCV treatment was significantly associated with having a high proportion of HCV-infected patients (>20% versus less <20%; OR 3.9; 95% confidence interval [CI] 1.5–10.0) and availability of other services at the primary care site including HIV treatment (OR 6.5 (2.5–16.5)), substance abuse treatment (OR 3.3 (1.3–8.4)) and mental health services (OR 4.9 (2.0–12.1)) (Table 3).

## Discussion

In this sample of primary care providers in a region with high HCV prevalence [9,17–19], a large proportion of PCPs were aware of new HCV treatments and agreed that treatment would benefit the communities they serve. However, only 22% of PCPs agreed that in the era of interferon-free, direct-acting regimens, HCV treatment should be provided by PCPs, and they were statistically more likely to have a high prevalence of HCV and have patient support services in their clinics. As one of the first studies to assess willingness among community-based PCPs to directly treat patients with HCV, this study underscores the importance of addressing provider reluctance in expanding HCV treatment. The findings also suggest the largest short-term impact might be achieved by concentrating efforts on those with a high prevalence of HCV infection among their patients and support services within their clinic.

PCPs have been widely considered critical to expanding access to HCV treatment for the large number of individuals infected with HCV in the US [6,10]. Although the vast majority of hepatitis C care will likely remain within the realm of specialty care, PCPs trained in HCV care could help alleviate workforce shortages in relation to HCV care and treat patients in their primary care home where linkage to specialty care has been a barrier. Operationalization of PCP driven hepatitis C care, however, requires a better understanding of facilitators and barriers to HCV care provision by PCPs. In our study we found that having a large primary care patient load was a significant barrier to willingness to provide HCV therapy. This suggests that PCPs who already likely feel overwhelmed with provision of care for multiple other medical problems of a large number of patients are less likely to be willing to add on HCV treatment to their patient care portfolio.

Interestingly, PCPs who either had a large proportion of their patients infected with hepatitis C or had access to support services for populations disproportionately affected by hepatitis C, such as those with HIV infection and substance abuse, were more likely to be willing to provide hepatitis C treatment. While we did not specifically ask why PCPs were willing or not willing to provide treatment, this may reflect either that these providers perceive HCV to be a higher priority among their patients or have a level of experience and comfort with this patient population which makes taking on treatment of a disease that disproportionately affects these populations less of a burden.

It was encouraging that the majority of PCPs in our cohort were aware of CDC defined “birth cohort” based hepatitis C screening recommendations and were screening in their clinical practice. As expected, few PCPs were treating HCV. As such, it is not surprising that self-rated HCV care proficiency was, on average low among providers in this cohort. Given the high rates of alcohol use comorbidity in HCV-infected individuals and the negative impact of alcohol use on liver related health in HCV-infected individuals [20,21], it is reassuring that self-rated alcohol counseling proficiency was high among PCPs. The US Centers for Disease Control and Prevention (CDC) recommends that persons identified as having HCV infection should receive a brief screening for alcohol use and intervention. Despite high alcohol use counseling proficiency, the majority of PCPs did not identify alcohol use counseling as part of initial management of a patient recently diagnosed with hepatitis C, consistent with findings from a previous study [14]. This further illustrates the need for additional HCV care training among PCPs. Outside direct HCV treatment, PCPs have high value in HCV care provision given ready access and opportunities to provide harm reduction messages such as alcohol use screening, brief intervention and referral to treatment as indicated on an ongoing basis. These alcohol use interventions provided by PCPs would be especially cost effective as most PCPs may not require additional alcohol use counseling training [22].

It is interesting to note that despite low willingness of PCPs to provide HCV treatment, there was high interest in additional HCV training. Interest in additional HCV training was not associated with perceived self-efficacy, suggesting that HCV knowledge is a general area of interest for PCPs. PCPs were also willing to gain this knowledge through a number of avenues including through on-line modules such as those provided by the University of Washington [23], sessions at conferences and local CME workshops. Organizations providing education to primary care providers should consider including training opportunities for HCV in the education opportunities provided. Even for providers who choose not to treat HCV, additional training may improve rates of HCV screening and appropriate ongoing counseling, including alcohol use counseling and other harm reduction strategies.

## Limitations

Our study is limited by the small sample size and the localization of providers to one urban region of the United States. The findings may not be generalizable to rural regions where increasingly HCV infection is found among younger persons who inject drugs. In addition, we were unable to assess bias in our convenience sample of respondents due to lack of information about individuals who refused participation. Our response rates are in line with other provider surveys and suggest validity of our findings.

Another limitation of this study is the use of a questionnaire. While questionnaires can be effective in measuring behaviors, attitudes, preferences, and opinions, they may create limitations. In this instance, the fixed-choice questionnaire assumed an understood general knowledge, obliging the respondents to answer questions through a narrow scope.

## Conclusion

To the extent that provider attitudes predict their receptivity to changes in clinical practice, these data suggest that the largest HCV treatment impact would be achieved by training care providers at sites with many HCV patients and by concurrently creating clinical support systems and time to facilitate the additional effort.

## Figures and Tables

**Table 1:** Provider demographic and practice site characteristics.

Provider Characteristic	No. (%)	Practice site characteristics	No. (%)
Provider Type		Practice location‡	
MD/DO	84 (70)	Urban	58 (48)
NP/PA	36 (30)	Suburban	60 (50)
Gender		Rural	1 (<1)
Female	82 (68)	Academic affiliation	
Male	38 (32)	No	101 (84)
Age		Yes	19 (16)
<36 years	26 (22)	Percent of patients PWID‡	
36–45 years	51 (43)	<10%	69 (58)
46+ years	43 (36)	Nov-25	20 (17)
Race		>25	20 (17)
White	72 (60)	Unsure	7 (6)
Asian	24 (20)	Percent of patients HCV infected	
Black	18 (15)	<20%	91 (76)
Other*	6 (5)	>20%	25 (21)
Years practicing median (IQR), years	5 (2–13.5)	Unsure	4 (3)
Number of patients cared for in prior 12 months		Percent of patients with public insurance	
<100	11 (9)	<25%	39 (32)
101–500	20 (17)	26–50%	27 (22)
501–1000	27 (22)	51–75%	29 (24)
>1000	62 (52)	> 75%	25 (21)

PWID- People who inject drugs; IQR- Interquartile range;

* 2 individuals identified as Latino, 2 South Asian and 2 declined to report their race;

‡ Practice location not reported by 1 individual, percent of patients PWID not reported by 4 individuals

**Table 2:** Provider self-rating of HCV care proficiency.

None	Limited Skills No. (%)	Average among peers No. (%)	Very Knowledgeable No. (%)	Expert No. (%)
Ability to assess severity of liver disease				
3 (3)	27 (24)	63 (55)	21 (18)	0
Ability to identify candidates suitable for HCV treatment				
3 (3)	26 (23)	63 (55)	22 (19)	0
Ability to educate and motivate hepatitis C patients				
2 (2)	26 (23)	54 (47)	27 (24)	5 (4)
Ability to treat HCV and manage therapy side effects				
24 (21)	64 (56)	20 (17)	6 (5)	0
Ability to choose HCV treatment regimens using current guidelines				
34 (30)	53 (46)	20 (17)	7 (6)	0
**Alcohol and substance use care proficiency**				
Ability to provide a brief alcohol screen, counseling and referral for alcohol use				
2 (2)	11 (10)	48 (42)	44 (39)	9 (8)
Ability to assess for and manage substance use comorbidities				
4 (3)	25 (22)	54 (47)	27 (24)	4 (3)

**Table 3:** Predictors of PCP willingness to provide HCV treatment.

Provider Characteristic	Odds Ratio	Clinical site characteristics	Odds Ratio
Provider type		% of patients HCV infected*	
MD/DO	1	<20%	1
NP/PA	2.5 (0.9–5.0)	>20%	3.9 (1.5–10.0)
Sex*		% of patients IDU	
Female	1	<25	1
Male	2.5 (1.0–6.1)	>25%	1.6 (0.6–4.7)
Age*		Onsite services	
>45	1	Substance use*	
36–45	3.3 (1.3–10)	No	1
<36	2.0 (0.7–5.0)	Yes	3.3 (1.3–8.4)
No of patients seen in prior 12 months*		HIV treatment*	
<100 patients	1	No	1
101–1000 patients	0.2 (0.04–0.7)	Yes	6.5 (2.5–16.5)
> 1000 patients	0.1 (0.02–0.4)	Mental health*	
HCV proficiency score		No	1
<10points	1	Yes	4.9 (2.0–12.1)
> 10 points	0.8 (0.3–2.2)		

* Statistically significant at P<0.05
